# Antimicrobial and Morphological Effect of Er,Cr:YSGG Laser Irradiation on Primary Dentine Infected by Microorganisms Associated With Early Childhood Caries

**DOI:** 10.3290/j.ohpd.c_2315

**Published:** 2025-10-22

**Authors:** Cesar Abraham Sandoval-Marcelino, Bernardo Teutle-Coyotecatl, Gerardo Landeta-Cortés, Flor de Lourdes Arriaga-Lila, María del Pilar Martín-Santiago, Rosalía Contreras-Bulnes, Laura Emma Rodríguez-Vilchis, Rosario Jiménez-Flores, Estela del Carmen Velasco-León, María de los Angeles Moyaho-Bernal

**Affiliations:** a Cesar Abraham Sandoval-Marcelino Student, Faculty of Stomatology, Meritorious Autonomous University of Puebla, Mexico. Performed the experiments in partial fulfilment of requirements for a degree.; b Bernardo Teutle-Coyotecatl Professor, Department of Pediatric Dentistry, Faculty of Stomatology, Meritorious Autonomous University of Puebla, Mexico. Wrote the manuscript.; c Gerardo Landeta-Cortés Professor, Dirección de Innovación y Transferencia de Conocimiento (DITCo), Meritorious Autonomous University of Puebla, Mexico. Performed testing.; d Flor de Lourdes Arriaga-Lila Associate Professor, Mac Hospital, Puebla, Mexico. Proofread the manuscript.; e María del Pilar Martín-Santiago Professor, University of La Laguna, Spain. Proofread the manuscript.; f Rosalía Contreras-Bulnes Professor, Center for Research and Advanced Studies in Dentistry, School of Dentistry, Autonomous University of Mexico State, Mexico. Proofread the manuscript.; g Laura Emma Rodríguez-Vilchis Professor, Center for Research and Advanced Studies in Dentistry, School of Dentistry, Autonomous University of Mexico State, Mexico. Proofread the manuscript.; h Rosario Jiménez-Flores Professor, Faculty of Stomatology, Meritorious Autonomous University of Puebla, Mexico. Wrote the manuscript.; i Estela del Carmen Velasco-León Professor, Department of Pediatric Dentistry, Faculty of Stomatology, Meritorious Autonomous University of Puebla, Mexico. Proofread the manuscript.; j María de los Angeles Moyaho-Bernal Professor, Department of Pediatric Dentistry, Faculty of Stomatology, Meritorious Autonomous University of Puebla, Mexico. Wrote the manuscript.

**Keywords:** antimicrobial effect, Er, Cr:YSGG laser, Lactobacillus, Candida albicans

## Abstract

**Purpose:**

Laser technology enables a less stressful lesion removal with antimicrobial effects, this study was addressed to evaluate the antimicrobial and morphological effect of Erbium, Chromium: Yttrium-Scandium-Gallium-Garnet (Er,Cr:YSGG) laser on infected primary dentine with *Lactobacillus* spp. and *Candida albicans*.

**Materials and Methods:**

*In-vitro* experimental study, where 35 samples of primary dentine were randomly distributed into 5 groups (n = 7): 3 controls (non-irradiated G1_C, G2_LB and G3_CA) and 2 experimental (G4_LB+Er,Cr:YSGG and G5_CA+Er,Cr:YSGG). The samples were placed in culture media pre-inoculated with *Lactobacillus* spp. and *C. albicans*, according to the experimental group, and maintained under optimal temperature and sterility conditions; then groups G4_LB+Er,Cr:YSGG and G5_CA+Er,Cr:YSGG were irradiated with Er,Cr:YSGG laser at 4.5 w, 15 Hz, 1 s/mm^2^ (H mode) and 53.6 J/cm^2^. Subsequently, they were transferred to sterile culture media, and microbial colony-forming units (CFUs) were performed in triplicate after 24 h using serial dilutions. The adherent bacteria and morphology of primary dentine were observed with scanning electron microscopy (SEM).

**Results:**

There were significant differences among control groups (G2_LB and G3_CA) and experimental (G4_LB+Er,Cr:YSGG and G5_CA+Er,Cr:YSGG) groups (P < 0.001); additionally, the percentage reduction after laser treatment was 99% for group G4_LB+Er,Cr:YSGG and 98% for G5_CA+Er,Cr:YSGG, indicating a significant reduction. On the other hand, samples irradiated with laser Er,Cr:YSGG [G4_LB + Er,Cr:YSGG], [G5_CA + ErCr:YSGG] showed an irregular surface with areas exhibiting micro-erosion and microcavities.

**Conclusions:**

Er,Cr:YSGG laser irradiation presented an antimicrobial effect against *Lactobacillus* spp. and *C. albicans*. Moreover, this procedure can alter the dentinal surface structure.

Early childhood caries (ECC) is a chronic disease characterised by the presence of one or more decayed, noncavitated or cavitated lesions, missing due to caries, or filled tooth surfaces in any primary tooth of a child under the age of 6 years. It presents a rapid development of caries, affecting several teeth after their eruption in the oral cavity.^1^

According to the latest report from the National Epidemiological Surveillance System of Oral Pathologies in Mexico, 73.2% of children have early childhood caries.^20^ Worldwide, ECC affects between 35–70% of children, especially in low- and middle-income countries.^27^

In caries development, several microorganisms are involved; *Streptococcus mutans* participates in the early stages of the disease, while *Lactobacillus* spp. are associated with the progression of caries in the enamel and dentin. Likewise, studies have demonstrated the presence of *C. albicans* in children suffering from ECC.^16^

Within the pediatric dentistry area, the incorporation of new technologies is becoming more common, with a marked preference for those that turn out to be minimally invasive. Dental laser is a good example, as it is an instrument with great and ideal versatility to perform treatments in children and adolescents.^17,19^

Some of the most important properties of lasers that are used in dentistry are its ablative power, bactericidal effect and an excellent acceptance by the pediatric patients. The interaction between the laser and tissue is a biologic effect that depends on the optic properties of the tissue.^5^

The Erbium, Chromium: Yttrium-Scandium-Gallium-Garnet (Er,Cr:YSGG) laser (2780 nm) is a high-power class IV medium infrared laser, which emits its wavelength in a pulsed mode.^9^ It is equipped with an aerosol of water and air, which, combined with the laser beam, cools the zone of incidence, minimising the collateral thermal effects and increasing its mechanism of action. This laser also has a good absorption of water and hydroxyapatite, and is frequently used in surgical procedures on bone tissue and soft tissues of the oral cavity, including the removal of restorations. Finally, it produces decontaminated surfaces by eliminating carious tissue, anaerobic and aerobic bacteria, due to tissue ablation with an antibacterial effect.^5,9^

Some laser equipment has been previously studied. The photothermal and mechanical effects of Er:YAG laser were evaluated to reduce cariogenic species concentration, demonstrating a significant difference in terms of total microbial load reduction compared with the use of conventional therapy.^9, 24^ On the other hand, laser diode was investigated to assess the effect of photodynamic therapy on multispecies oral caries biofilms composed of *S. mutans*, *Lactobacillus casei*, and *C. albicans*. The results supported the positive effects of photodynamic therapy on the reduction of all three microorganisms. Specifically, *C. albicans* was significantly reduced on the biofilm.^7^ Additionally, the use of photodynamic therapy has been demonstrated to be a good alternative or complementary treatment to conventional antimicrobial therapies and has an antifungal effect against *C. albicans*.^13^

With the continuous evolution and development of equipment applied to dentistry, dental lasers are one of the most innovative and promising technologies. Therefore, the objective of this study is to evaluate the antimicrobial and morphological effect of Er,Cr:YSGG laser irradiation on primary dentine infected with microorganisms associated with ECC.

## MATERIAL AND METHODS

### Tooth Selection and Sample Preparation

The protocol was reviewed and approved by the Research Committee of the Faculty. All teeth donors signed a consent form. The procedures were done in accordance with the Declaration of Helsinki (2013). Nine primary molars extracted because of prolonged retention without decay, fluorosis, fractures, or fillings were collected. Immediately after extraction, teeth were stored in a 0.2% thymol solution and transported to the laboratory. The specimens were cleaned with distilled water, and traces of soft tissue were removed with a scalpel. The crowns were then gently brushed with a soft brush (Sulcus, Oral-B, Mexico), and finally rinsed with triply distilled water. Samples were stored at 4°C in a 0.2% thymol solution before the analysis.^23^

The crowns of primary molars were then rinsed with triply distilled water and air-dried. Then, a mesiodistal central cut was performed using a low-speed diamond wheel saw (South Bay Technology, USA) under constant distilled water irrigation. Four dentine square blocks (3 × 3 mm) were obtained, two from the buccal and two from the lingual surface. Subsequently, they were dried at room temperature, and observed with a light microscope (AXIO ZEIZZ Scope. A1, Germany) to confirm enamel absence at 35×. The dentine block was defined as the experimental unit. The samples were then randomly assigned to five groups, each containing seven dentine units:

G1_C: samples of non-inoculated dentine.
G2_LB: samples of dentine inoculated with *Lactobacillus* spp.
G3_CA: samples of dentine inoculated with *C. albicans*.
G4_LB+Er,Cr:YSGG: samples of dentine inoculated with *Lactobacillus* spp. and later irradiated with Er,Cr:YSGG laser.
G5_CA+Er,Cr:YSGG: samples of dentine inoculated with *C. albicans* and later irradiated with Er,Cr:YSGG laser.

**Fig 1:**
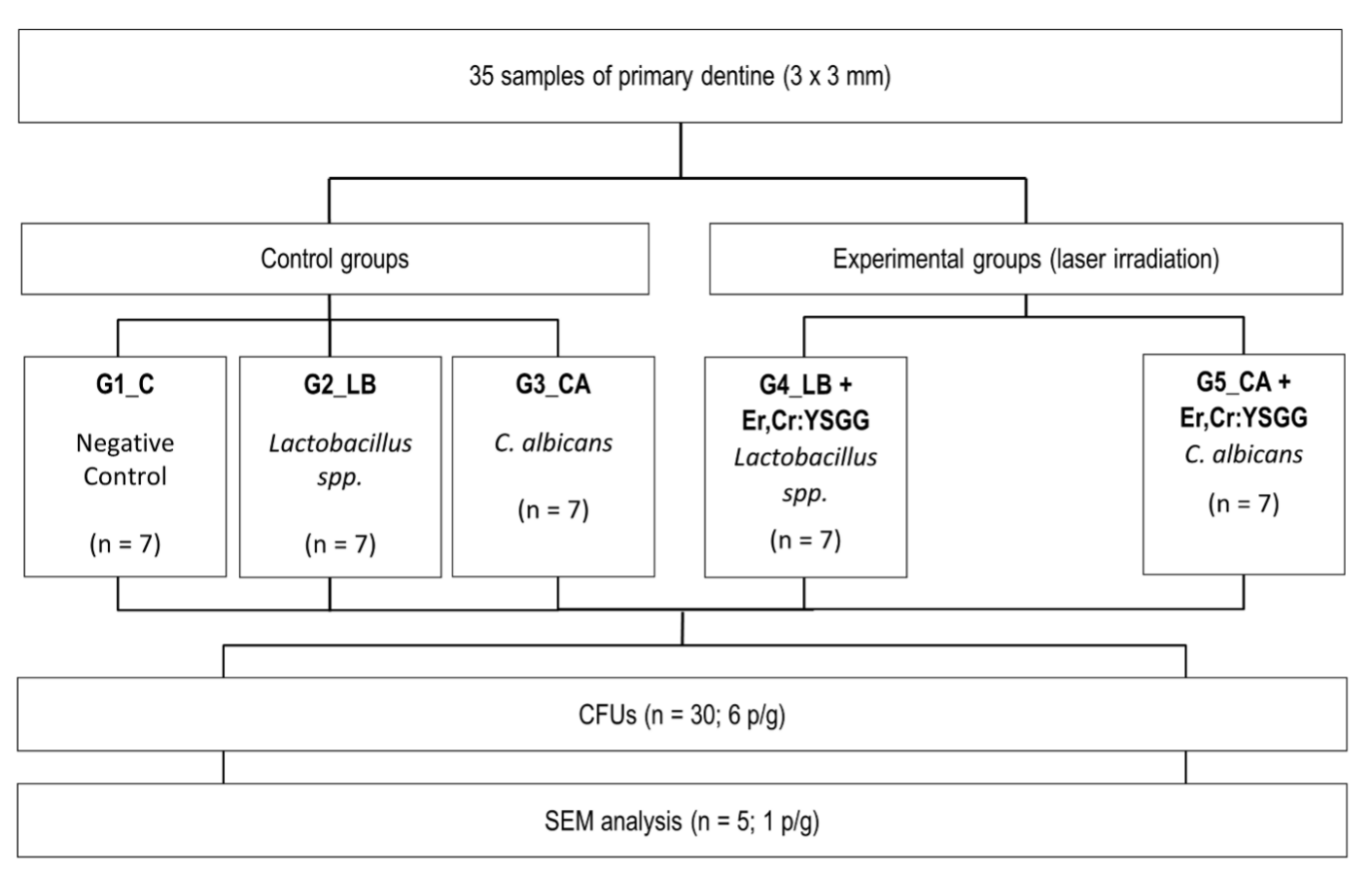
Procedures and techniques.

The sequence of the procedures and techniques applied in this study are shown in Figure 1.

### Strain Isolation and Recovery

The microorganisms *Lactobacillus* spp. and *C. albicans* were isolated from children with ECC. *Lactobacillus* spp. was inoculated on the MRS agar (De Man, Rogosa, and Sharpe) in microaerophilic conditions: on the other hand, C. albicans was cultivated on CHROM agar and after that on Luria-Bertani agar (LB) in aerophilic conditions at 37°C for 24 h. After that, the purity of the cultures was verified through morphological tests.

### Identification

First, Gram staining was performed to identify whether the bacterium is Gram-positive and to visualise the morphology of the bacterial cell. It should be emphasised that bacteria were isolated using a selective culture medium that favours their growth; in particular, Man, Rogosa, and Sharpe (MRS) agar is used for lactic acid bacteria.^8^

Similarly, Gram staining was performed to observe *C. albicans*, which appears as spheres or blastoconidia in the form of ‘bubbles’. A chromatic specificity medium was used, which allows the growth of different *Candida* species and has specific indicators to facilitate the identification of *C. albicans*.^28^

### Preparation of Microbial Suspensions

Single isolated cells of *Lactobacillus spp*. and *C. albicans* were allowed to grow for 24 h at 37°C in 9 mL of MRS (microaerophilic conditions) and LB broth medium (aerophilic conditions), respectively. Bacterial and yeast suspensions were standardised to 0.5 McFarland, with an approximate equivalence of 1–2 × 10^-8^ cells/mL.^6^

### Inoculation

Prior to the bacterial adhesion test, the samples of primary dentine were washed for 10 min with deionised water in an ultrasonic bath (Quantrex Q140, L & R Ultrasonics, NJ, USA) and then placed in an Eppendorf tube (Eppendorf Safe-Lock®, Eppendorf SE Germany) containing 1 mL of deionised water to carry out sterilisation at 120 °C for 15 min.^23^

All experiments were performed using 14 samples per bacterium and 7 per group. Sterile dentine samples were individually placed in Eppendorf tubes (Eppendorf Safe-Lock®, Eppendorf SE Germany) with the previously determined medium for the five different groups. They were kept in suspension at room temperature for 30 min. Afterwards, the samples were removed from the tube and under optimal sterile and temperature conditions, the excess culture was removed and irradiated with the Er,Cr:YSGG laser.

### Er,Cr:YSGG Laser Irradiation

An Er,Cr:YSGG laser system [Er Cr: YSGG (Waterlase iPlus Biolase, CA, USA)] was employed for sample irradiation. The parameters used were a wavelength emission of 2.78 µm, 4.5 W, 15 Hz, and 1 s/mm^2^ (H mode). The laser beam was delivered in a non-contact and non-focused mode. The total time of irradiation was 9 s, under deionised water irrigation (5 mL/min) at 80% and 30% air. An energy pulse of 53.6 J/cm^2^ was employed with a MZ8 tip.^25,29^

To promote uniform irradiation, the tip was positioned perpendicular to the dentine surface, and the irradiation was performed manually in one direction with a consistent motion. The irradiation tip-sample distance was standardised at 1 mm. The energy levels were calibrated with a device provided by the manufacturer and were monitored periodically with a power meter (LaserMate-P, Coherent Co., Santa Clara, CA). A laminated infrared sensor screen (Lumitek International, Ijamsville, MD) was employed to verify that the exit tip and the laser beam had the same diameter.^23^

### Colony-Forming Units (CFUs)

After laser irradiation, the samples were placed in a sterile broth of MRS (DifcoTM, CONEPRE, Mexico) and LB (MilliporeSigma, Merck KGaA, USA). Then, they were left in suspension for 30 min and resuspended with the help of a vortex (Wisemix-10, WITEG Labortechnik, Germany) for their homogenisation. Subsequently, from the culture medium, the serial dilutions procedure was carried out, taking 1 mL of the culture medium with the sample and diluting it in a sterile culture medium. The procedure was successively repeated. Each of the dilutions obtained was plated in the proper culture media and conditions in triplicate, obtaining a final n = 90. All the petri dishes (polystyrene petri dish – SYM, CTR SCIENTIFIC, Mexico) were incubated for 24 h at 37°C to count the CFUs.

### Scanning Electron Microscopy

An extra sample of each surface was prepared to observe the adherent bacteria with SEM following the standard procedures described above. The samples were stabilised in 1mL of a 2% glutaraldehyde solution for 1 h and dehydrated in serial dilutions (20, 40, 60, 80, 100% v/v) of ethanol for 20 min. Subsequently, the samples were air-dried for one day and then transferred to aluminium stubs and coated with gold (160s, 40 mA). The scanning electron microscopic evaluation took place in an SEM JEOL (JSM-6610 LV, EU) at 20 kV.^22,23^

### Statistical Analysis

Data were analysed using the SPSS (Statistical Package for Social Science) programme, version 22 (SPSS IBM., New York, NY, USA). The data distribution was evaluated by the Shapiro–Wilk test; then, the Student’s t-test for independent samples between groups was used to establish comparisons of the CFUs to the same species adhered to the surfaces; a P < 0.05 significance threshold was used.

## RESULTS

### CFUs

The mean and reduction rate in CFUs (%) of control (G2_LB and G4_LB) and irradiated groups (G4_LB + Er,Cr:YSGG and G5_CA + Er,Cr:YSGG) are shown in Table 1. The group G1_C did not present any colony growth. Student’s t-test revealed a significant difference among G2_LB vs G4_LB + Er,Cr:YSGG and G3_CA vs G5_CA + Er,Cr:YSGG group (P < 0.001). Additionally, reduction percentages were evaluated. The groups G4_LB + Er,Cr:YSGG and G5_CA + Er,Cr:YSGG showed 99.9% and 98.7 % reductions in CFUs, respectively.

**Table 1:** Average and standard deviation of *Lactobacillus* spp. and *C. albicans* bacterial cells adhered to primary dentine with and without laser irradiation

Groups	Mean CFUs × 10^3^	Reduction in CFUs (%)
Control	G1_C	–	±	–		
G2_LB	419.83	±	242.97	**A**	
G3_CA	230.6	±	148.9	**A**	
Experimental	G4_LB + Er,Cr:YSGG	1.7	±	1.3	**B**	**99.9**
G5_CA + Er,Cr:YSGG	3	±	1.9	**B**	**98.7**
* Capital letters in a column compare the same bacteria between the control and experimental groups. The same letters mean that they do not differ statistically (Student’s t-test for independent samples between groups; P >0.05).

### SEM Observations

**Fig 2:**
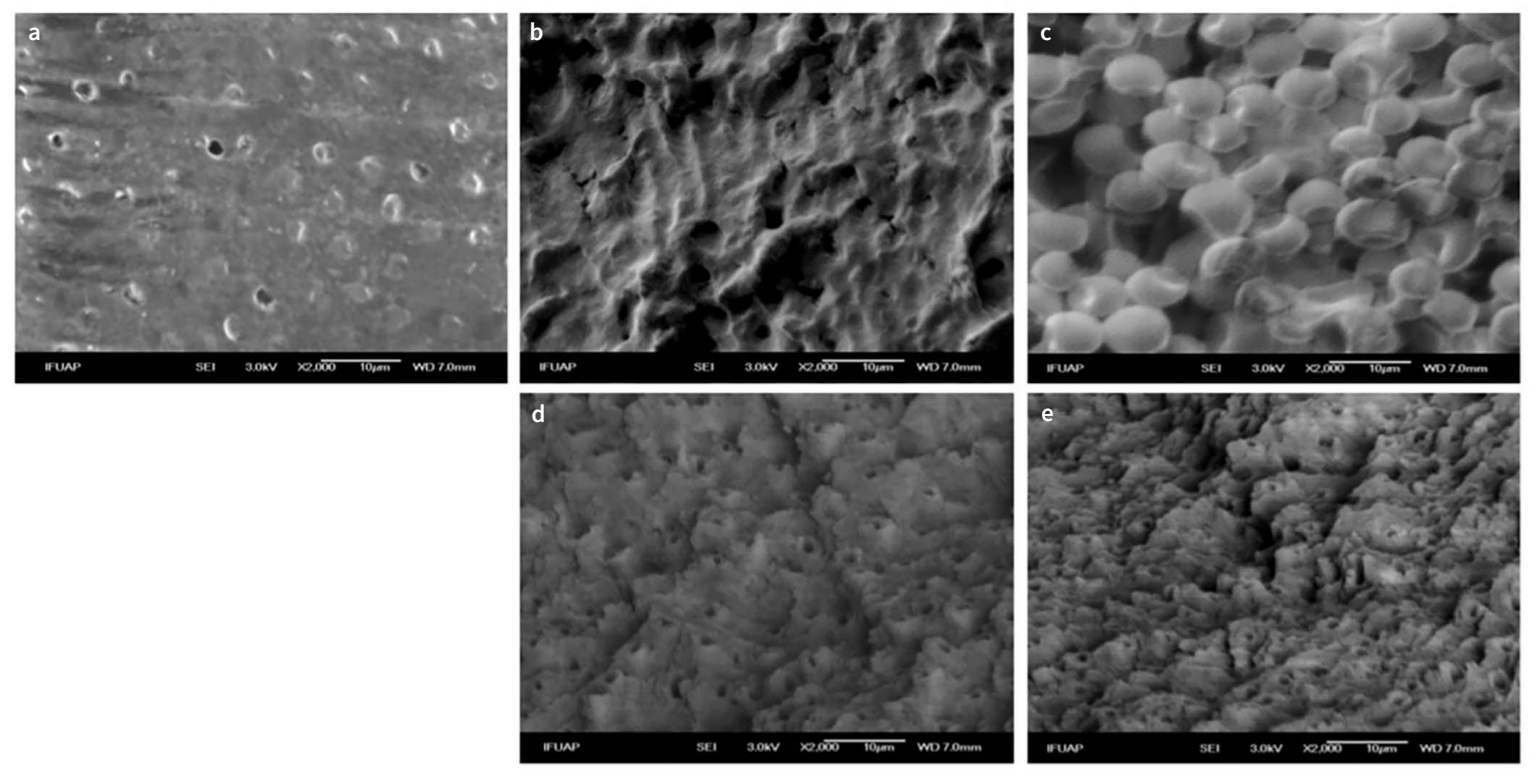
Representative scanning electron microscopic (SEM) images of experimental groups. (a) Dentine without microorganisms inoculation; (b) dentine with *Lactobacillus* spp.; (c) dentine with *C. albicans*; (d) dentine with *Lactobacillus* spp. and Er,Cr:YSGG laser irradiation; (e) dentine with *C. albicans* and Er,Cr:YSGG laser irradiation.

The dentine surface of the study groups is depicted in Figure 2. Smooth dentine surfaces and some grooves and exposed dentine tubules were observed on untreated dentine of the negative control group [G1_C] (Fig 2a).

The group inoculated with *Lactobacillus* spp. [G2_LB] showed the presence of these microorganisms on the dentine surface and close to dentine tubules (Fig 2b). The group inoculated with *C. albicans* [G3_CA] displayed similar characteristics, presenting fungus on the dentine surface (Fig 2c). Additionally, the dentine inoculated with bacteria or fungus and irradiated with Er,Cr:YSGG [G4_LB + Er,Cr:YSGG], [G5_CA + ErCr:YSGG] presented a rough surface and more exposed dentine tubules; as well as absence of microorganisms (Fig 2d and 2e).

## DISCUSION

Currently, laser technology has become an alternative to the use of conventional therapies for the treatment of early childhood caries and disinfection. This research evaluated the antimicrobial effect of the Er,Cr:YSGG in dentine infected by *Lactobacillus* spp. and *C. albicans*. Similar results were observed, like those reported by other authors in similar studies using lasers from the Erbium family. The use of the Er,Cr:YSGG laser in operative dentistry has several aspects that affect its efficacy and clinical applicability. This laser, possessing a wavelength of 2780 nm, is highly absorbed by hard tissues, such as enamel and dentine, which is fundamental for a precise therapeutic action.^2^

The hydrokinetic energy method of disinfection is particularly noteworthy, as it involves the efficient removal of the smear layer, a contaminant layer formed during the development of a carious lesion. This process not only reduces the bacterial load significantly, but also improves the bond strength values of the adhesive used in dental restorations.

The Er,Cr:YSGG laser offers significant advantages over traditional tooth preparation methods, such as rotary drills. For example, its ability to create a cleaner, contaminant-free working environment can result in improved clinical outcomes and a reduction in the incidence of recurrent caries in permanent dentition.^3^ This is particularly important in modern pediatric dentistry, where the preservation of tooth tissue and improvement in the quality of restorations are increasingly sought.

According to recently published research works, the effects of laser irradiation on the dentine surface can be modified in relation to the parameters used. In irradiated surfaces there is an increase in roughness, changes in chemical composition, among other things, which give additional benefits such as a laser etching pattern, that could improve the adhesion and disinfect surfaces at the same time.^4^

In this investigation, the laser irradiation demonstrated a significant reduction for both microorganisms: *Lactobacillus* spp. (with a reduction of 99.9%) and *C. albicans* (showing a reduction of 98.7%) with statistically significant differences among the non-irradiated samples and inoculated with microorganisms after the aforementioned. These results are in line with what was reported in literature, where Baraba et al showed that the effect of the Er:YAG, at different pulsations, can eliminate carious dentine and the present cariogenic bacteria.^2^ Even more, Valenti et al demonstrated a significant reduction for both microorganisms using Er:YAG, presenting a total reduction of *Lactobacillus* spp. and a 94.4% one in *C. albicans*.^7^

In agreement with the findings of Valenti et al, who investigated the effect of laser on the reduction of microorganisms in carious dentine, a 99.9% reduction was observed in this study for the *Lactobacillus* spp. count after Er,Cr:YSGG laser treatment.^7^ This significant reduction in bacterial load suggests that the laser is highly effective in eliminating *Lactobacillus* spp. from infected primary dentine.^2^

It is known that the Er,Cr:YSGG light presents minimally invasive properties due to its high selectivity. Within target cells, there may be molecules or molecular species termed chromophores. A chromophore is defined as ‘a chemical group (molecule or molecular species) that absorbs light’.^15^ In addition, this laser is absorbed by the hydroxyl ion (OH^–^). The effect of light absorbed by the chromophores and hydroxyl ions is manifested in the conversion to thermal energy and ablation of the microorganism by photothermolysis (increase of temperature). The water of the microorganism and its surface is heated, expanding it and causing microexplosions, which end up killing it.^18^ This process can explain the antimicrobial effect achieved by Er,Cr:YSGG laser irradiation.

In this research work, wild strains isolated from patients with ECC were used because they have a greater capacity to adapt to the environment, better adhesion to surfaces, and augmented expression of virulence factors.^23^

Additionally, the present study analysed the qualitative adhesion of *Lactobacillus* spp. and *C. albicans* on Er,Cr:YSGG laser-treated and untreated dentine using SEM to visualise morphological and structural changes on the dentine surface.

The intact dentine samples [G1_C] presented a smooth surface and some exposed dentine tubules without the presence of any microorganisms on the surface. These same observations were reported in the work of Wang et al where they show images of a smooth primary dentine surface with some exposed tubules.^26^

On the other hand, the samples inoculated with *Lactobacillus* spp. [G2_LB] and *C. albicans* [G3_CA] showed the presence of these microorganisms scattered all over the surface. The samples inoculated with *Lactobacillus* spp. displayed a greater amount of these microorganisms compared to C. albicans. Fungus is an opportunistic human pathogen that can grow as a yeast, pseudohypha or true hypha. Depending on the conditions of the environment in which it is found, this will determine its pathogenesis and pathogenicity; additionally, *Lactobacillus* spp. presents a smaller structure and a better adaptability to the environment, although less resistance in comparison to the fungus.^10,11^

Likewise, the groups irradiated with laser Er,Cr:YSGG [G4_LB + Er,Cr:YSGG], [G5_CA + ErCr:YSGG] showed an irregular surface with areas exhibiting micro-erosion and microcavities, indicative of laser ablation in a selective manner. These findings are in line with previous studies that have shown that lasers can modify the surface structure of dentine, creating a cleaner surface and removing biological and structural contaminants.^10^ In the surfaces irradiated, no bacterial or fungal cells were seen due to the Er,Cr:YSGG laser antimicrobial properties, since it generates hydrokinetic energy and heat on the dentine surface, both of which can affect the viability and proliferation of bacteria and fungi. The use of laser not only involves the physical elimination of microorganisms present in infected dentin, but may also alter the structure of the dentine surface, creating less favourable conditions for future bacterial colonisation.^12,14,21^

It is important to consider that this study was performed *in vitro*. Furthermore, the efficacy of the Er,Cr:YSGG laser may vary depending on the specific treatment conditions, such as the energy dose applied, the duration of irradiation, and the microorganisms present. Therefore, future research should continue to explore these aspects of clinical protocols to further optimise their antimicrobial efficacy and evaluate their long-term safety in clinical treatments, particularly when working with paediatric patients.

## CONCLUSIONS

The Er,Cr:YSGG laser irradiation of primary dentine demonstrated a statistically significant reduction of *Lactobacillus* spp. and *C. albicans*. Additionally, this procedure can alter the dentinal surface structure.
